# Residual Derivative-Guided Spectral Fusion Module for Few-Shot Classification of Soybean Seed Varieties Using Hyperspectral Imaging

**DOI:** 10.3390/foods15152663

**Published:** 2026-07-29

**Authors:** Xiaoyu Fu, Guoyi Yu, Kai Gao, Qinfeng Zhang, Wenjie Liu, Lei Zhou, Chu Zhang, Chenchen Xue, Lu Huang

**Affiliations:** 1 School of Information Engineering, Huzhou Normal University, Huzhou 313000, China; 2 College of Mechanical and Electronic Engineering, Nanjing Forestry University, Nanjing 210037, China; 3 Institute of Industrial Crops, Jiangsu Academy of Agricultural Sciences, Nanjing 210014, China

**Keywords:** soybean seed, variety classification, hyperspectral imaging, few-shot learning, residual spectral fusion

## Abstract

Soybean seed variety identification is essential for seed quality control, germplasm management, and variety authentication. However, few-shot classification remains challenging because different varieties often exhibit highly similar one-dimensional hyperspectral signatures, and labeled samples are limited in practical seed-testing scenarios. This study proposes a Residual Derivative-Guided Spectral Fusion (RDSF) module to improve spectral representation under limited-sample conditions. RDSF uses the raw spectrum and its first- and second-order derivatives to characterize global reflectance patterns, local slope variations, and spectral curvature, respectively. The three representations are processed by separate branches and combined through bounded learnable residual fusion, with the raw spectrum serving as the primary representation and the derivatives providing complementary corrections. As a plug-and-play component, RDSF was integrated into Prototypical Network (ProtoNet), Relation Network (RelationNet), and Model-Agnostic Meta-Learning (MAML). The module was evaluated using spectra from 11,000 individual soybean seeds representing 11 varieties under known-class and strict class-disjoint unseen-class protocols. Under the representative known-class 3-way 10-shot setting with 15 query samples per class, RDSF increased the meta-test accuracy of RelationNet from 0.8898 ± 0.0201 to 0.9184 ± 0.0060. Under the unseen-class protocol, RDSF consistently improved ProtoNet and RelationNet across all evaluated shot settings; the largest gain was observed for ProtoNet in the 5-shot setting, with the meta-test accuracy increasing from 0.8848 ± 0.0253 to 0.9094 ± 0.0080. In contrast, RDSF did not consistently improve MAML under this protocol, indicating that its effectiveness depended partly on the underlying meta-learning mechanism. Ablation experiments and architecture comparisons further showed the complementary contributions of the derivative branches and the advantages of bounded residual fusion over a three-channel architecture and direct feature concatenation. Overall, RDSF provides an effective spectral representation module for metric-based few-shot classification of soybean seed varieties under the evaluated known-class and unseen-class conditions.

## 1. Introduction

Soybean is an important food, oil, and plant-protein crop worldwide, with major roles in food processing, animal feed production, plant-protein supply, and agricultural economies [1,2]. Reliable identification of soybean seed varieties is essential for seed quality control, germplasm management, and variety authentication. Conventional identification methods include manual morphological inspection, physicochemical analysis, and molecular marker-based detection. Although these methods can be reliable, they may be subjective, time-consuming, labor-intensive, costly, or partially destructive, limiting their suitability for rapid and large-scale screening. Therefore, developing an efficient and non-destructive method for soybean seed variety identification is of practical importance for modern seed industry management and precision agriculture.

Hyperspectral imaging (HSI) records continuous spectral responses over a broad wavelength range and enables rapid, non-destructive, and information-rich analysis [3]. Unlike conventional red–green–blue (RGB) imaging, which records reflected light in three broad visible channels and primarily describes color and spatial characteristics, HSI captures wavelength-dependent differences that may not be visually distinguishable [4]. It has consequently been applied to seed quality evaluation, viability assessment, disease detection, origin discrimination, and variety classification [5]. In this study, the mean spectrum extracted from each segmented seed was used as a one-dimensional input rather than the full spectral–spatial image cube. This representation retains the wavelength-resolved reflectance response of an individual seed while reducing input complexity, providing a practical basis for rapid seed-level classification.

HSI-based classification generally relies on machine learning or deep learning to extract discriminative information from high-dimensional spectral data [6,7,8]. Models ranging from support vector machines (SVMs), random forests (RF), and partial least squares discriminant analysis (PLS-DA) to convolutional neural networks (CNNs), residual networks (ResNets), long short-term memory networks (LSTMs), and Transformers have been applied to spectral classification [9,10,11]. Although these supervised approaches can perform well when representative labeled data are available [12], their reliability generally depends on the quantity and quality of the training samples. Because seed collection, hyperspectral image acquisition, label confirmation, and balanced dataset construction require considerable time and labor, obtaining abundant labeled spectra for every variety may be impractical. Reliable classification with limited labeled samples therefore remains a key challenge in HSI-based soybean seed variety identification.

Few-shot learning (FSL) addresses this limitation by classifying query samples from a small support set containing only a few labeled examples per class [13]. Meta-learning is a major FSL paradigm in which models are trained through episodic tasks to acquire transferable strategies for constructing task-specific classifiers [14]. Prototypical Network (ProtoNet) represents each class by a prototype in the embedding space [15], whereas Relation Network (RelationNet) learns a nonlinear relation function between class and query representations [16]. Model-Agnostic Meta-Learning (MAML), by contrast, learns an initialization that can be adapted to a new task through a small number of gradient updates [17]. Few-shot performance can be evaluated under two distinct protocols. In the known-class protocol, meta-training, meta-validation, and meta-testing share the same label space but use nonoverlapping seed samples, thereby assessing limited-sample classification of predefined varieties. In the strict class-disjoint protocol, the classes used at the three stages are mutually exclusive, thereby assessing adaptation to varieties absent from meta-training. The former addresses classification with limited reference samples for known varieties, whereas the latter evaluates generalization to unseen varieties.

Although recent studies have applied few-shot and meta-learning methods to HSI classification [18,19,20], many were developed for spectral–spatial image patches and do not directly address representation learning from one-dimensional seed spectra. Standard implementations of ProtoNet, RelationNet, and MAML also typically encode the input spectrum using a single backbone. Although such encoders can learn wavelength-dependent patterns, they do not explicitly separate the overall reflectance profile from local slope and curvature variations. The raw spectrum preserves the complete reflectance response, whereas its first- and second-order derivatives emphasize local slope and curvature, respectively. These representations may reveal subtle spectral-shape differences between varieties with similar raw spectra. However, because derivative spectra are deterministic transformations of the raw spectrum, they may also amplify local fluctuations, particularly at the second order. Therefore, an effective fusion strategy is needed to exploit their complementary information while preserving the complete raw spectral profile.

To address this representation problem, this study proposes a Residual Derivative-Guided Spectral Fusion (RDSF) module for the few-shot classification of soybean seed varieties. RDSF processes the raw, first-order derivative, and second-order derivative spectra through separate branches and projects them into a common embedding space. The resulting features are integrated through residual fusion to combine the complementary information provided by the three spectral representations. RDSF is designed as a plug-and-play representation module rather than an independent classifier and can be integrated into different meta-learning frameworks without altering their episodic training and testing procedures.

The main contributions of this study are summarized as follows:

(1) An RDSF module is proposed to enhance representation learning from one-dimensional hyperspectral spectra under few-shot conditions. It explicitly incorporates global reflectance, local slope, and curvature information through raw, first-order derivative, and second-order derivative representations.
(2) A residual derivative-guided fusion mechanism is developed to integrate the raw, first-order derivative, and second-order derivative representations. This design enables derivative features to supplement the raw spectral information within a unified embedding space.
(3) RDSF is integrated into ProtoNet, RelationNet, and MAML and evaluated under complementary known-class and strict class-disjoint unseen-class protocols. This design examines its behavior across metric-based and optimization-based frameworks while distinguishing limited-sample classification of predefined varieties from generalization to unseen varieties.
(4) Comprehensive experiments are conducted using spectra from 11,000 individual seeds representing 11 soybean varieties. Performance is evaluated across different shot settings and task difficulties, while ablation experiments, controlled architecture comparisons involving three-channel input and direct feature concatenation, fusion coefficient analysis, episode-level stability analysis, and MAML adaptation diagnostics provide complementary evidence regarding the effectiveness, stability, and framework dependence of RDSF.

## 2. Materials and Methods

### 2.1. Sample Preparation

A total of eleven soybean seed varieties were used in this study, namely Haimen Baiyouguozi, Qidong Xianhaodou, Danyang Wanchadou, Haimen Dengxifeng, Yangzhou Sanhuang, Haimen Huangke, Xinyi Heikezi, Nantong Yuandou, Rudong Popi, Tongshan Niumaohuang, and Nanjing Gaoxiangdou. Before hyperspectral image acquisition, damaged, shriveled, broken, moldy, and visually abnormal seeds were manually excluded. After screening, 1000 intact seeds were retained for each variety, resulting in a total of 11,000 individual seed samples. Seeds from different varieties were stored and processed separately according to their variety labels. Before imaging, the seeds were placed on the imaging platform with sufficient spacing to prevent overlap or adhesion between adjacent seeds and to facilitate subsequent individual-seed segmentation. Each soybean seed was treated as an independent sample.

### 2.2. HSI System and Hyperspectral Image Acquisition

The HSI system used in this study consisted of a hyperspectral camera, a mobile platform, a white reference panel, a lighting system, and a computer equipped with Lumo Scanner 2020 software (Spectral ImagingLtd., Oulu, Finland) for image acquisition. The system was operated in line-scan imaging mode. The hyperspectral camera (FX17, Spectral Imaging Ltd., Oulu, Finland) acquired images in the near-infrared range of 900–1700 nm with 224 spectral bands. A standard white reference panel made of polytetrafluoroethylene (PTFE) was used for reflectance calibration.

Two sets of halogen lamps were used as the illumination source. Each set contained three lamps, and the power of each lamp was 35 W. The lamps were symmetrically positioned on both sides of the camera to provide stable and uniform illumination during image acquisition. Before acquisition, the exposure time and the moving speed of the mobile platform were adjusted to obtain clear and unsaturated hyperspectral images. In this study, the moving speed of the mobile platform was set to 24.7 mm/s, and the exposure time was set to 8.26 ms.

**Figure 1:**
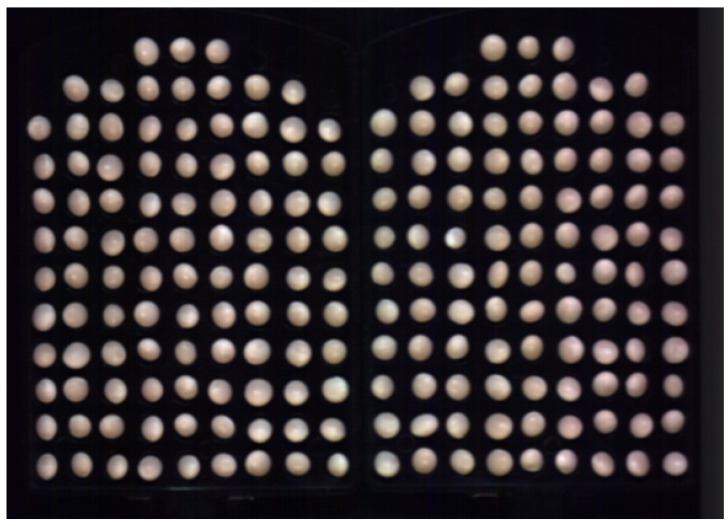
A representative pseudo-color image generated from the hyperspectral image of soybean seed samples.

Soybean seeds were placed in seed-counting trays for hyperspectral image acquisition, with 100 seeds arranged in each tray. Two trays were placed in parallel on the moving platform and scanned simultaneously. This arrangement improved imaging efficiency while ensuring that individual seeds were spatially separated and clearly identifiable in the hyperspectral images. Figure 1 shows a representative pseudo-color image generated from the hyperspectral image of soybean seed samples.

To reduce the effects of dark current and uneven illumination, dark- and white-reference correction was applied to the raw hyperspectral images. The corrected reflectance image was calculated as follows:

(1) $$R=\frac{R_{0}-B}{W-B},$$

where $R_{0}$ represents the raw hyperspectral image, B represents the dark reference image obtained by covering the camera lens, W represents the white reference image measured using the standard PTFE white reference panel, and R represents the corrected reflectance image. The corrected hyperspectral images were then used for subsequent seed segmentation and spectral information extraction.

### 2.3. Spectral Extraction and Dataset Preparation

The grayscale image corresponding to the 100th spectral band was selected for seed segmentation because it provided clear contrast between the soybean seeds and the background. Threshold-based binarization was applied to generate a binary mask, and connected-component analysis was subsequently used to identify and label individual seed regions. The mask corresponding to each detected seed was applied to the corrected hyperspectral cube. For each spectral band, the mean reflectance of an individual seed was calculated by averaging all seed pixels within the corresponding region. The resulting mean reflectance values across all spectral bands were exported as comma-separated values (CSV) files to construct the original spectral dataset.

Because hyperspectral imaging is sensitive to instrument performance, illumination conditions, and environmental factors, spectral bands at the beginning and end of the wavelength range often contain relatively strong noise and redundant information. Therefore, the first 10 bands and the last 14 bands were removed, and the remaining 200 consecutive bands, corresponding to Bands 11–210, were retained as the raw spectral inputs for subsequent analysis. Before calculating the derivative spectra, a five-point moving average was applied to each retained spectrum as a simple short-window smoothing method to suppress residual high-frequency fluctuations.

After spectral extraction and band removal, all soybean seed spectra were organized into datasets for supervised learning and few-shot learning experiments. For the supervised-learning experiments and the known-class few-shot evaluation, the extracted spectra were divided into training, validation, and test sets using a ratio of 3:1:1. The dataset was stratified according to soybean variety to maintain the same class distribution in each subset. Specifically, 6600 spectra were used for training, 2200 spectra were used for validation, and 2200 spectra were used for testing. The training, validation, and test sets shared the same 11-class label space but contained no overlapping seed samples. To reduce the influence of a single data partition, five random number generator seeds (18, 35, 57, 69, and 82) were used to generate five independent sample-level partitions. All models under the supervised-learning and known-class few-shot protocols were trained, validated, and tested using the same five partitions. The class-disjoint unseen-class evaluation instead used mutually exclusive class groups for meta-training, meta-validation, and meta-testing.

### 2.4. Algorithm

#### 2.4.1. Conventional Supervised Models

To establish supervised baselines for soybean seed variety classification, seven models were implemented using the extracted one-dimensional hyperspectral data: support vector classifier (SVC), CNN, ResNet, Transformer, LSTM, CNN–Transformer, and CNN-LSTM [21]. All models were trained and evaluated using the same training, validation, and test partitions described in Section 2.3. Their results were used to assess the general separability of the soybean seed spectra and provide reference performance for the subsequent few-shot learning experiments.

SVC was used as the conventional machine-learning baseline. It constructs a decision boundary by maximizing the margin between classes and is effective for classifying high-dimensional spectral data [22]. A radial basis function (RBF) kernel was adopted to model nonlinear relationships among the hyperspectral features. The penalty parameter C and kernel parameter gamma were optimized through grid search over the range $\left[10^{-7},\;10^{7}\right]$, combined with 10-fold cross-validation conducted exclusively on the training set. The parameter combination yielding the highest cross-validation accuracy was used to refit the SVC on the full training set, after which its performance was evaluated on the training, validation, and test sets.

CNN was used to learn local spectral patterns and dependencies among adjacent wavelength bands [23]. The model contained two one-dimensional convolutional blocks followed by three fully connected layers. The two convolutional layers used 128 and 64 filters, respectively, with a kernel size of 5. Each convolutional layer was followed by batch normalization and rectified linear unit (ReLU) activation. The extracted features were flattened and passed through fully connected layers with output dimensions of 256, 128, and 11, with the final layer producing the classification logits for the 11 soybean varieties. The model was trained for 1000 epochs using the cross-entropy (CE) loss and the Adam optimizer, with a batch size of 64, a learning rate of 0.0005, and a weight decay of 0.0001.

ResNet was adopted to evaluate residual learning for one-dimensional hyperspectral classification. Its shortcut connections facilitate gradient propagation and alleviate network degradation as the model depth increases [24]. A one-dimensional ResNet-18 architecture was implemented, beginning with a convolutional stem comprising 64 filters, a kernel size of 7, a stride of 2, batch normalization, ReLU activation, and max pooling. Four residual stages with channel dimensions of 64, 128, 256, and 512 were subsequently used, with each stage containing two basic residual blocks. The extracted features were processed by adaptive average pooling and an 11-dimensional fully connected classification layer. The model was trained for 1000 epochs using the CE loss and the Adam optimizer, with a batch size of 32, a learning rate of 0.00001, and a weight decay of 0.0001.

Transformer was used as an attention-based baseline for modeling global dependencies among spectral bands [25]. Unlike convolution, which primarily captures local spectral patterns, self-attention establishes interactions among different wavelength positions. Each 200-dimensional spectrum was represented as a sequence of 200 positions with one feature channel, and a linear projection mapped each spectral value into a 64-dimensional embedding. Positional encoding was added to retain wavelength-order information. The Transformer encoder contained two encoder layers with four attention heads. The encoded sequence was averaged along the spectral dimension and passed through a fully connected layer for 11-class classification. The model was trained for 1000 epochs using the CE loss and the AdamW optimizer, with a batch size of 32, a learning rate of 0.0001, and a weight decay of 0.0001.

LSTM was employed to model sequential dependencies along the ordered wavelength dimension [26]. Each spectrum was represented as a sequence of length 200 with one feature channel. Before training, the spectra were standardized using the mean and standard deviation calculated exclusively from the training set, and the resulting parameters were subsequently applied to the validation and test sets. The LSTM contained two recurrent layers with a hidden dimension of 256. The output at the final time step was passed through an 11-dimensional fully connected layer to generate the classification logits. The model was trained for 1000 epochs using the CE loss and the Adam optimizer, with a batch size of 16, a learning rate of 0.0003, and a weight decay of 0.0001. A step learning-rate scheduler with a step size of 100 epochs and a decay factor of 0.9 was used.

CNN–Transformer was used to combine local spectral feature extraction with global attention-based modeling [27]. Its CNN block contained two one-dimensional convolutional layers with 128 and 64 filters, respectively; both layers used a kernel size of 5 and padding of 2, followed by batch normalization and ReLU activation. The resulting feature sequence was projected into a 128-dimensional embedding space and processed by a Transformer encoder containing two encoder layers with four attention heads. The encoded sequence was averaged along the spectral dimension and passed through fully connected layers with output dimensions of 64 and 11. The model was trained for 1000 epochs using the CE loss and the AdamW optimizer, with a batch size of 32, a learning rate of 0.0001, and a weight decay of 0.0001.

CNN-LSTM was used to combine local spectral feature extraction with sequential dependency modeling. Its CNN block contained two one-dimensional convolutional layers with 128 and 64 filters, respectively; both layers used a kernel size of 5 and padding of 2. The resulting feature maps were rearranged as a spectral sequence and passed to a two-layer LSTM with a hidden dimension of 128. The final hidden representation was processed by fully connected layers with output dimensions of 64 and 11 to generate the classification logits. The model was trained for 1000 epochs using the CE loss and the Adam optimizer, with a batch size of 64, a learning rate of 0.0001, and a weight decay of 0.0001.

#### 2.4.2. Few-Shot Learning Task Formulation

FSL was used to evaluate soybean seed variety classification when only a few labeled reference samples were available for each class. Unlike conventional supervised learning based on a fixed classification task, FSL employs episodic learning, in which each episode constitutes an independent classification task containing a support set and a query set. The support set provides limited labeled samples for constructing task-specific classification information, whereas the query set is used to calculate the training objective or evaluate classification performance. Two complementary protocols were considered. In the known-class protocol, meta-training, meta-validation, and meta-testing shared the same 11 soybean variety labels but used nonoverlapping seed samples. This protocol evaluated limited-sample classification of predefined varieties. In the strict class-disjoint protocol, mutually exclusive varieties were assigned to meta-training, meta-validation, and meta-testing to evaluate generalization to varieties absent from meta-training.

Each episode was formulated as an N-way K-shot Q-query task. For each episode, N soybean varieties were selected from the class subset assigned to the corresponding stage. For every selected variety, K labeled samples were randomly selected to form the support set, and Q additional samples were selected to form the query set. The support and query sets were defined as:

(2) $$S=\left\{\left(x_{n,k}^{s},y_{n}\right)\left|n=1,\;\ldots ,\;N;k=1,\;\ldots ,\;K\right.\right\},$$

(3) $$Q=\left\{\left(x_{n,q}^{q},y_{n}\right)\left|n=1,\;\ldots ,\;N;q=1,\;\ldots ,\;Q\right.\right\},$$

where S and Q denote the support and query sets, respectively; $x_{n,k}^{s}$ and $x_{n,q}^{q}$ represent their one-dimensional hyperspectral samples; and $y_{n}$ denotes the corresponding class label. Each episode therefore contained N×K support samples and N×Q query samples. The original variety labels were remapped to local episode labels ranging from 0 to N−1, and no seed sample appeared in both sets within the same episode.

During meta-training, the support set was used to construct task-specific classification information or perform task-specific adaptation, and the learnable parameters were optimized using the query-set loss. For a given episode, the CE loss was calculated as:

(4) $$L_{CE}(\theta )=-\frac{1}{NQ}\sum_{n=1}^{N}\sum_{q=1}^{Q}\log p_{\theta }\left(y_{n}x_{n,q}^{q},S\right),$$

where $p_{\theta }\left(y_{n}x_{n,q}^{q},S\right)$ denotes the predicted probability that query sample $x_{n,q}^{q}$ belongs to class $y_{n}$, conditioned on the support set, and θ represents the learnable model parameters. During meta-validation and meta-testing, episodes were independently sampled from the corresponding data subsets using the same task formulation. The query samples were not included in support-based inference or adaptation, and their labels were used only to calculate the validation objective or final classification performance.

Three groups of few-shot experiments were conducted. First, the effect of the support-set size and the behavior of RDSF across different meta-learning frameworks were evaluated under the known-class 3-way 5-shot 15-query, 3-way 10-shot 15-query, and 3-way 15-shot 15-query settings. ProtoNet, RelationNet, MAML, and their RDSF-enhanced versions were compared using the same episodic configurations.

Second, the effect of task difficulty was evaluated under the known-class 3-way, 5-way, and 11-way settings, with the support and query sizes fixed at 10 and 15 samples per class, respectively. The 3-way episodes contained 30 support and 45 query samples, the 5-way episodes contained 50 support and 75 query samples, and the 11-way episodes contained 110 support and 165 query samples. Thus, “15-query” denotes 15 query samples per class rather than 15 samples in the entire episode. Using the same query size for every class ensured equal class contributions and consistent task definitions while maintaining a manageable computational cost in the 11-way experiments. The 3-way and 5-way settings assessed discrimination among subsets of varieties, whereas the 11-way setting included all varieties and represented a more challenging full-class task.

Third, strict class-disjoint experiments were conducted to evaluate generalization to unseen soybean varieties. The 11 varieties were assigned class indices from 0 to 10 according to their order in Section 2.1 and divided into five meta-training classes (Classes 0–4), three meta-validation classes (Classes 5–7), and three meta-test classes (Classes 8–10). No variety was shared among the three subsets. Each meta-training episode sampled three of the five training classes, whereas all three assigned classes were included in each meta-validation and meta-test episode. The support and query samples were sampled independently without overlap. This protocol was evaluated under the 3-way 5-shot 15-query, 3-way 10-shot 15-query, and 3-way 15-shot 15-query settings. Model selection used only episodes from the meta-validation classes; the meta-test classes were used exclusively for final evaluation after model selection. Consequently, the meta-test results reflected generalization to classes absent from meta-training rather than episodic resampling within a shared label space.

Within each evaluation protocol, baseline and RDSF-enhanced models used the same data partitions or class assignments, task configurations, and episodic sampling strategy. This controlled design ensured that their performance differences were evaluated under consistent experimental conditions.

#### 2.4.3. Baseline Meta-Learning Frameworks

Three representative meta-learning frameworks were implemented: ProtoNet, RelationNet, and MAML. ProtoNet and RelationNet represent metric-based approaches, whereas MAML represents optimization-based meta-learning. All three followed the episodic task formulation described in Section 2.4.2. Their baseline implementations and corresponding RDSF-enhanced versions used the same sample partitions and episodic settings under the known-class protocol and the same class assignments under the strict class-disjoint protocol.

ProtoNet learns an embedding space in which samples are classified according to their distances from class prototypes. In each episode, a shared spectral encoder mapped the support and query samples into the embedding space. The embeddings of the K support samples belonging to each class were averaged to construct its prototype, and each query was classified using its Euclidean distances from the N prototypes. The negative distances were treated as classification logits. The baseline encoder contained two one-dimensional convolutional blocks and a linear projection layer. The first convolutional layer used 128 filters with a kernel size of 5 and a stride of 1, followed by batch normalization, ReLU activation, and max pooling. The second layer used 64 filters with the same kernel size and stride, followed by the same operations. The flattened features were projected into a 128-dimensional embedding space. ProtoNet was trained using the query-set CE loss and the Adam optimizer with a learning rate of 0.0001.

RelationNet replaces a predefined distance metric with a trainable relation module that learns the similarity between class-level support representations and query representations. In each episode, a shared encoder first generated support and query embeddings. The support embeddings of each class were averaged, concatenated with each query embedding, and passed through the relation module to produce one relation score for each class–query pair. These scores were used as the classification logits. The baseline encoder comprised a one-dimensional convolutional layer with 256 filters, a kernel size of 5, and a stride of 3, followed by batch normalization and ReLU activation. The resulting features were flattened, passed through a fully connected layer with 128 neurons, and projected into a 64-dimensional embedding space. The relation module contained three fully connected layers: the first two had 128 hidden units, with ReLU activation applied after each hidden layer, and the final layer produced a single relation score. RelationNet was trained using the query-set CE loss and the Adam optimizer, with a learning rate of 0.001 and a weight decay of 0.000001.

MAML learns a shared initialization that can be adapted to an episode-specific classifier using a small support set. For each episode, the model was adapted on the support set through inner-loop gradient updates and subsequently evaluated on the query set. The post-adaptation query loss was then used to optimize the shared initialization in the outer loop. The baseline spectral backbone comprised one one-dimensional convolutional layer with 128 filters, a kernel size of 5, and a stride of 3, followed by instance normalization and ReLU activation. The flattened features were passed through a fully connected layer with 128 neurons and projected into a 64-dimensional feature space. A task-specific linear classification head was constructed in each episode, with an input dimension of 64 and an output dimension equal to N. The inner loop used stochastic gradient descent with a learning rate of 0.01 and two update steps. The outer loop used Adam with a meta-learning rate of 0.00001 and a meta-batch size of 32. Full second-order MAML was implemented rather than its first-order approximation.

A post hoc diagnostic analysis was conducted to examine the comparatively lower performance of MAML under the known-class 3-way 10-shot 15-query setting. The five independently trained MAML models corresponding to the five dataset partitions were evaluated using 200 fixed meta-validation episodes. Adaptation trajectories were examined at 0, 1, 2, 3, 5, 7, and 10 inner-loop steps, with the learning rate fixed at 0.01. Within each episode, the support and query samples and task-head initialization remained identical across the evaluated step numbers. Support accuracy, meta-validation query accuracy, and their difference were recorded. Gradient consistency at initialization was quantified using cosine similarity between the support and meta-validation query gradients for both the complete task model and the spectral backbone. The coverage of the 165 possible class combinations and the normalized Shannon entropy of their sampling frequencies were calculated.

#### 2.4.4. Proposed RDSF Module

To enhance spectral representation under limited labeled samples, this study proposed an RDSF module that uses the raw spectrum (Raw), first-order derivative spectrum (D1), and second-order derivative spectrum (D2) as three complementary inputs. Raw retains the overall reflectance profile, whereas D1 and D2 describe local slope and curvature variations, respectively. The three representations are processed through separate spectral branches and subsequently integrated using residual fusion.

For a soybean seed sample, the raw spectral vector is denoted as follows:

(5) $$X_{Raw}=\left[x_{1},\;x_{2},\;\ldots ,\;x_{L}\right],$$

where $X_{Raw}$ represents the original one-dimensional hyperspectral vector, $x_{i}$ denotes the reflectance value at the i-th retained spectral band, and L is the number of retained spectral bands. In this study, L=200, and $X_{Raw}$ was used as the input to the Raw branch.

The D1 was calculated to describe local slope variations between adjacent spectral bands. It is defined as follows:

(6) $$D_{1}\left(i\right)=x_{i+1}-x_{i},\;\;i=1,\;2,\;\ldots ,\;L-1,$$

where $D_{1}\left(i\right)$ denotes the first-order derivative value between the i-th and (i+1)-th spectral bands. The five-point moving average described in Section 2.3 was applied before the derivative was calculated. Zero-padding was not used to maintain the 200-band length. Instead, the first valid adjacent-band difference was duplicated at the beginning of the D1 vector. Consequently, the first two D1 values were identical, preventing a large artificial discontinuity at the spectral boundary.

The D2 was calculated from D1 to describe curvature variations. It is defined as follows:

(7) $$D_{2}\left(i\right)=D_{1}\left(i+1\right)-D_{1}\left(i\right),\;\;i=1,\;2,\;\ldots ,\;L-1,$$

where $D_{2}\left(i\right)$ denotes the second-order derivative value calculated from two adjacent D1 values. The same boundary-value duplication procedure was applied to D2. Because the first two D1 values were identical, the initial boundary difference in D2 was zero. Therefore, no zero-padding or large artificial edge was introduced, and Raw, D1, and D2 retained the same length of 200.

RDSF contained three branch-specific one-dimensional convolutional encoders. Each branch comprised two convolutional layers with a kernel size of 5 and a stride of 1, and each layer was followed by batch normalization and ReLU activation. The Raw branch used 128 and 64 output channels in the first and second convolutional layers, respectively. The D1 branch used 32 and 32 channels, whereas the D2 branch used 16 and 16 channels. The Raw branch was assigned a larger convolutional capacity because it retained the complete reflectance profile, while the derivative branches were used to extract complementary slope and curvature features. Each branch output was subsequently flattened and independently projected into a 64-dimensional embedding space.

Let $f_{Raw}$, $f_{D1}$, and $f_{D2}$ denote the feature extraction branches for Raw, D1, and D2, respectively. The corresponding feature embeddings are expressed as follows:

(8) $$F_{Raw}=f_{Raw}\left(X_{Raw}\right),$$

(9) $$F_{D1}=f_{D1}\left(D_{1}\right),$$

(10) $$F_{D2}=f_{D2}\left(D_{2}\right),$$

where $F_{Raw}$, $F_{D1}$, and $F_{D2}$ denote the embeddings extracted from Raw, D1, and D2, respectively. All three branches produced 64-dimensional embeddings, enabling element-wise residual fusion without increasing the final embedding dimension.

The final RDSF embedding is obtained through residual spectral fusion:

(11) $$F_{RDSF}=F_{Raw}+\alpha_{1}F_{D1}+\alpha_{2}F_{D2},$$

where $F_{RDSF}$ denotes the fused spectral embedding, and $\alpha_{1}$ and $\alpha_{2}$ are the learnable fusion coefficients for D1 and D2, respectively. The coefficient of the Raw branch was fixed at 1.0 and was not learnable. The D1 and D2 coefficients were initialized to 0.10 and 0.05, respectively, and constrained to the range of 0–0.30 during optimization. The resulting 64-dimensional fused embedding was used as the spectral representation for subsequent few-shot classification.

RDSF was implemented as a plug-and-play representation module by replacing the original spectral encoder of each baseline framework while retaining its classification mechanism. In RDSF-ProtoNet, the fused embeddings were used to construct class prototypes and calculate query-to-prototype distances. In RDSF–RelationNet, the fused support and query embeddings were processed by the original relation module. In RDSF-MAML, RDSF served as the shared spectral backbone and participated in task-specific inner-loop adaptation. For RDSF-ProtoNet and RDSF–RelationNet, $\alpha_{1}$ and $\alpha_{2}$ were globally learned parameters and remained unchanged during individual validation and test episodes. For RDSF-MAML, the two coefficients were included in the task-specific parameter set and could be updated during inner-loop adaptation.

**Figure 2:**
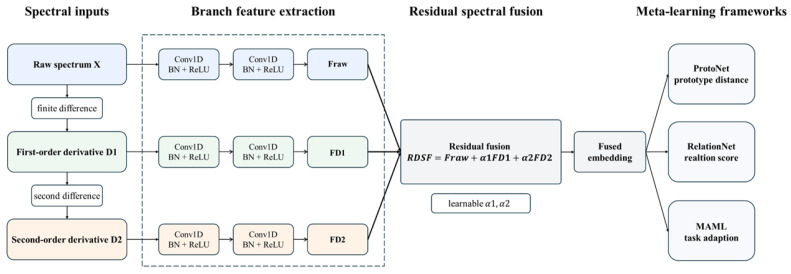
Architecture of the proposed RDSF module. The raw spectrum, first-order derivative spectrum, and second-order derivative spectrum are processed by separate spectral branches and integrated through residual spectral fusion. The fused spectral embedding can be used as a plug-and-play representation module for ProtoNet, RelationNet, and MAML.

To compare residual fusion with simpler integration strategies, two additional RelationNet variants were implemented under the known-class 3-way 10-shot 15-query setting. In ThreeChannel-RelationNet, Raw, D1, and D2 were stacked as three input channels and processed using a single shared convolutional backbone. In Concat-RelationNet, the three representations were processed using the same independent branches as RDSF, after which their 64-dimensional embeddings were concatenated into a 192-dimensional feature and projected back to 64 dimensions. RDSF–RelationNet retained the independent branches and integrated their embeddings using the residual formulation in Equation (11). The three variants were compared using the same five dataset partitions, episodic configuration, and model-selection procedure. Figure 2 presents the RDSF architecture and its integration with ProtoNet, RelationNet, and MAML.

### 2.5. Model Evaluation and Software Environment

Classification accuracy was used as the primary evaluation metric. For the conventional supervised models, Train ACC, Val ACC, and Test ACC denote the classification accuracies calculated on the training, validation, and test sets, respectively. The validation set was used exclusively for model selection, and the checkpoint with the highest validation accuracy was selected for final evaluation on the test set. Test-set performance was not used for parameter optimization or model selection. Classification accuracy was calculated as follows:

(12) $$Accuracy=\frac{N_{correct}}{N_{total}},$$

where $N_{correct}$ denotes the number of correctly classified samples and $N_{total}$ denotes the total number of evaluated samples.

For the meta-learning models, performance was evaluated through episodic testing. In each N-way K-shot Q-query episode, the support set was used to construct task-specific classification information or perform task-specific adaptation, after which the model predicted the labels of the query samples. The query accuracy of each episode was calculated as the proportion of correctly classified query samples. Train ACC, Val ACC, and Test ACC denote the mean query accuracies obtained from episodes sampled from the meta-training, meta-validation, and meta-test subsets, respectively. Train ACC was reported to characterize model fitting during episodic learning, Val ACC was used for checkpoint selection, and Test ACC represented final classification performance.

Under the known-class protocol, each selected checkpoint was evaluated on 200 independently sampled meta-test episodes in each dataset partition. The query size was fixed at 15 samples per class; therefore, each episode contained 15×N query samples under an N-way setting. Across the five independent sample-level partitions, each model–setting combination was evaluated on a total of 1000 meta-test episodes. Query accuracies were first averaged over the 200 episodes within each partition, and the resulting five partition-level mean accuracies were used for statistical reporting. Under the strict class-disjoint protocol, model selection was performed exclusively using episodes sampled from the meta-validation classes. After the best checkpoint had been selected, final performance was evaluated on 1000 meta-test episodes per run using the held-out meta-test classes. The class assignment was fixed, and five independent runs were conducted. Query accuracies were first averaged over the 1000 meta-test episodes within each run, and the resulting five run-level mean accuracies were used for statistical reporting.

The supervised-learning and known-class experiments used five independent sample-level partitions generated using the random number generator seeds 18, 35, 57, 69, and 82. The same partition associated with each random number generator seed was used for all compared models. The strict class-disjoint experiments used the fixed class assignment described in Section 2.4.2 and five independent runs initialized using the same five random number generator seeds. Accordingly, the supervised-learning and known-class results were summarized across five dataset partitions, whereas the strict class-disjoint results were summarized across five independent runs.

The mean accuracy and standard deviation were calculated as follows:

(13) $$Mean=\frac{1}{M}\sum_{m=1}^{M}{Acc}_{m},$$

(14) $$STD=\sqrt{\frac{\sum_{m=1}^{M}{\left({Acc}_{m}-Mean\right)}^{2}}{M-1}},$$

where M=5 denotes the number of independent dataset partitions or runs, and ${Acc}_{m}$ denotes the partition-level or run-level mean accuracy obtained from the m-th independent evaluation. The mean value reflects overall classification performance, whereas the standard deviation describes variability across the five independent evaluations.

For the post hoc MAML diagnostic analysis, each metric was first averaged over 200 fixed meta-validation episodes within each dataset partition and then summarized as mean ± standard deviation across the five partitions. Episode-level stability was evaluated separately from the standard final performance evaluation. Under the known-class 3-way 10-shot 15-query setting, the validation-selected checkpoints of RelationNet, RDSF-Raw, RDSF-Raw+D1, RDSF-Raw+D2, and RDSF–RelationNet were evaluated on 1000 fixed meta-test episodes per dataset partition. Identical episodes were used for all variants within each partition. Median accuracy, task-wise standard deviation, variance, interquartile range (IQR), and 10th-percentile accuracy were calculated within each partition and subsequently summarized across the five partitions.

All experiments were conducted on a 64-bit Windows 11 operating system. The hardware configuration included an Intel Core i7-14700KF CPU and an NVIDIA GeForce RTX 4070 Ti SUPER GPU with 16 GB of memory. Program development and execution were carried out using PyCharm 2025.1 and Python 3.12.9. The conventional machine learning model, namely SVC, was implemented using scikit-learn 1.6.0. The deep learning and meta-learning models, including CNN, ResNet, LSTM, Transformer, CNN-LSTM, CNN–Transformer, ProtoNet, RelationNet, MAML, and their RDSF-enhanced versions, were implemented using PyTorch 2.5.1 with CUDA 12.4.

## 3. Results

### 3.1. Spectral Profiles

**Figure 3:**
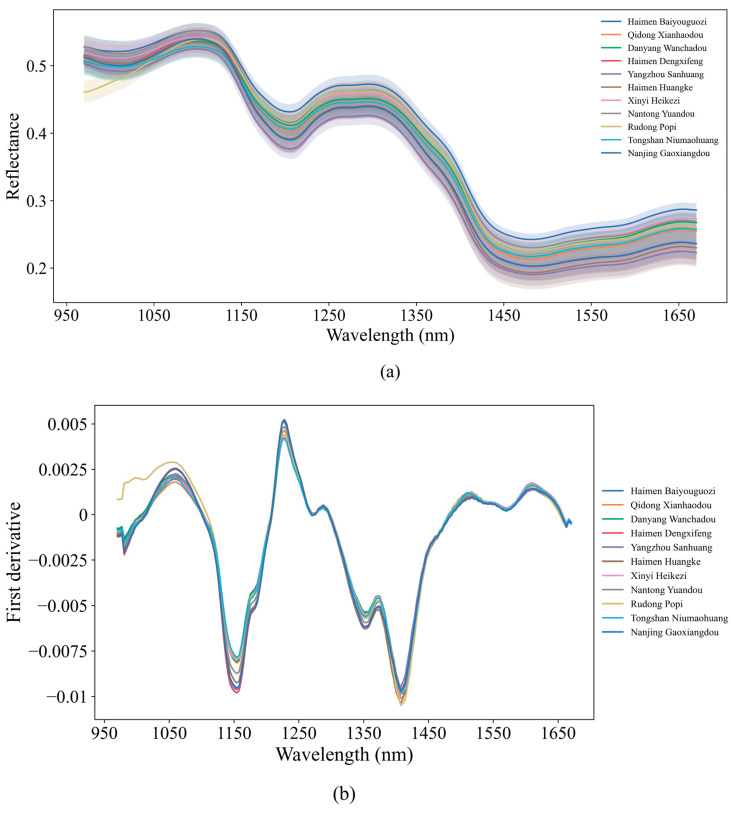

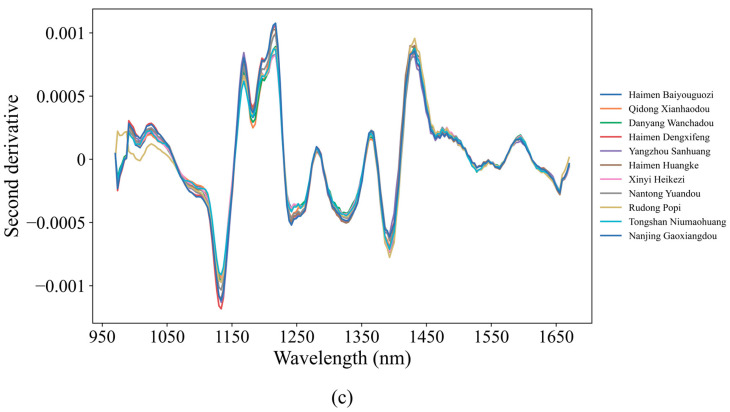
Comparison of the mean Raw, first-order derivative, and second-order derivative spectra of the 11 soybean seed varieties: (**a**) mean Raw reflectance spectra after radiometric correction, noisy edge-band removal, and five-point moving-average smoothing; (**b**) corresponding mean first-order derivative spectra; and (**c**) corresponding mean second-order derivative spectra. In (**a**), the shaded regions represent the within-variety standard deviations. The derivative panels show the mean curves without standard-deviation shading. The same color represents the same soybean variety across all three panels.

Figure 3 compares the mean Raw, D1, and D2 spectra of the 11 soybean varieties. As shown in Figure 3a, the Raw spectra exhibited broadly similar profiles across varieties. Reflectance remained relatively high in the short-wavelength region, decreased markedly around 1150–1200 nm, varied gradually between approximately 1250 and 1350 nm, and showed a pronounced absorption valley around 1450 nm before increasing toward the longer-wavelength region. Intervarietal differences in reflectance intensity were most apparent around 1150–1250, 1400–1500, and 1550–1670 nm. Nevertheless, substantial overlap remained among the mean curves, indicating that the varieties shared similar overall spectral characteristics and were difficult to distinguish through visual inspection of the Raw spectra alone.

The absorption characteristics in these wavelength regions can be interpreted in the context of previously reported near-infrared band assignments. Absorption near 970 nm has been associated with an O–H stretching overtone related to moisture [28]. Bands around 1200 nm have mainly been attributed to the second overtone of C–H stretching and may reflect lipid- and carbohydrate-related aliphatic constituents [29,30]. Absorption around 1450–1460 nm has been associated with the first overtone of O–H stretching in water [28,31], whereas features near 1500 and 1670 nm have been linked to protein-related N–H vibrations and C–H overtones, respectively [29,30]. These assignments provide spectroscopic context for the observed profiles but do not demonstrate that the classification models relied on these specific wavelengths or constituents.

Figure 3b presents the mean D1 spectra of the 11 varieties. In contrast to the overall intensity patterns shown by the Raw spectra, D1 emphasized the direction and magnitude of local reflectance changes along the wavelength dimension. Pronounced positive and negative variations occurred mainly in regions where the Raw spectra changed rapidly, particularly around 1100–1250 and 1350–1500 nm. Although the D1 curves retained similar overall tendencies, differences in the magnitude and local shape of these variations were visible among several varieties, indicating that local slope information provided an additional representation of intervarietal spectral differences. Figure 3c shows the corresponding mean D2 spectra. D2 exhibited alternating positive and negative extrema around turning regions of the Raw and D1 curves, thereby emphasizing local curvature changes that were less apparent in the Raw spectra. Differences among varieties were visible in the amplitudes and local shapes of these extrema, particularly within the main transition regions. Compared with D1, D2 also showed greater sensitivity to small local fluctuations.

Taken together, Figure 3 shows that Raw describes the overall reflectance profile, whereas D1 and D2 provide complementary descriptions of local slope and curvature. Because D1 and D2 are deterministic transformations of Raw rather than independent information sources, these visual differences should not be interpreted as direct evidence of specific biochemical contributions or wavelength-level model attribution. Instead, the profiles provide descriptive support for jointly evaluating the three spectral representations and illustrate why discriminative modeling is required for soybean varieties with strongly overlapping spectral characteristics.

### 3.2. Performance of Conventional Supervised Models

Table 1 presents the classification performance of the conventional supervised models on the soybean seed hyperspectral dataset. Train ACC denotes the proportion of correctly classified samples in the training subset, Val ACC denotes the classification accuracy on the validation subset used for model selection, and Test ACC denotes the classification accuracy on the held-out test subset used for final performance evaluation. The differences among the evaluated models indicate that the one-dimensional soybean seed spectra contained discriminative information, although the effectiveness of extracting this information varied across model architectures.

CNN achieved the strongest overall performance, obtaining the highest Train ACC of 0.9644 ± 0.0078 and the highest Val ACC of 0.8923 ± 0.0054. Its Test ACC reached 0.8857 ± 0.0091, demonstrating that the local spectral representations learned by the convolutional layers remained effective on the held-out test data. SVC produced the numerically highest Test ACC of 0.8890 ± 0.0124, which was only 0.0033 higher than that of CNN. However, considering the training, validation, and test results together, CNN was regarded as the strongest overall supervised model because it led in two of the three evaluation subsets while maintaining a Test ACC comparable to that of SVC. The competitive performance of both models indicates that local convolutional representation and nonlinear kernel-based classification were suitable for the present one-dimensional spectral data.

Among the remaining models, ResNet achieved a Test ACC of 0.8186 ± 0.0124, followed by CNN–Transformer at 0.7900 ± 0.0121. Transformer and CNN-LSTM obtained Test ACC values of 0.7245 ± 0.0169 and 0.7224 ± 0.0356, respectively, while LSTM showed the lowest Test ACC of 0.6258 ± 0.0285. Thus, increasing architectural depth or introducing attention-based and recurrent sequence modeling did not translate into higher classification accuracy under the present dataset and training configurations. These results describe the relative suitability of the evaluated architectures for this task but do not imply that long-range or sequential spectral information is inherently uninformative.

Overall, the supervised results establish the baseline separability of the 11 soybean varieties from their one-dimensional hyperspectral spectra. CNN provided the strongest overall performance, whereas SVC achieved the highest numerical Test ACC. These results provide reference performance for the subsequent few-shot learning experiments.

### 3.3. Known-Class Few-Shot Classification Performance of Baseline Meta-Learning Models

Under the known-class evaluation protocol, Table 2 presents the few-shot classification performance of the baseline and RDSF-enhanced meta-learning models under the 3-way K-shot 15-query settings. Among the baseline meta-learning models, RelationNet achieved the best overall performance across the three shot settings. Under the 3-way 5-shot 15-query setting, RelationNet obtained a Test ACC of 0.9014 ± 0.0070, which was higher than those of ProtoNet and MAML. ProtoNet achieved a Test ACC of 0.8498 ± 0.0131, whereas MAML obtained a lower Test ACC of 0.7352 ± 0.0160. These results suggest that the learned relation metric was effective in distinguishing class–query relationships when only five labeled support samples were available for each soybean variety.

When the number of support samples increased to 10, RelationNet and ProtoNet showed relatively close test performance. RelationNet achieved a Test ACC of 0.8898 ± 0.0201, while ProtoNet achieved 0.8862 ± 0.0094. ProtoNet obtained a slightly higher Val ACC, whereas RelationNet achieved a slightly higher Test ACC, indicating that both metric-based frameworks remained competitive under this setting. In contrast, MAML achieved a Test ACC of 0.7421 ± 0.0253, which remained lower than those of the two metric-based models. Under the 3-way 15-shot 15-query setting, RelationNet again achieved the highest baseline Test ACC, reaching 0.9145 ± 0.0062. ProtoNet achieved a Test ACC of 0.8965 ± 0.0301, while MAML obtained 0.7260 ± 0.0238. Compared with the 5-shot setting, both RelationNet and ProtoNet showed improved test performance when more support samples were available, whereas MAML did not show a clear improvement trend under the predefined two-step inner-loop adaptation protocol.

Overall, the baseline few-shot results demonstrate that meta-learning models can perform soybean seed variety classification under limited labeled samples, but their performance differs substantially across frameworks. RelationNet showed the most consistently competitive performance among the three baseline models, achieving the highest baseline Test ACC in all three shot settings. Therefore, RelationNet was selected as the representative framework for further evaluation under different N-way task difficulties in Section 3.5.

### 3.4. Mechanistic Analysis of MAML Adaptation Behavior

Table 3 presents the inner-loop adaptation trajectory of MAML under the representative known-class 3-way 10-shot 15-query setting. This analysis was conducted post hoc using fixed meta-validation episodes and did not involve the meta-test data. Before adaptation, the support and meta-validation query accuracies were 0.3359 ± 0.0196 and 0.3350 ± 0.0174, respectively, both of which were close to the random accuracy of 0.3333 for a three-way classification task. After one inner-loop update, the query accuracy increased markedly to 0.6781 ± 0.0219, demonstrating that gradient-based adaptation could effectively extract task-specific information from the limited support samples. Under the original two-step configuration, the support and query accuracies further increased to 0.8036 ± 0.0162 and 0.7507 ± 0.0214, respectively.

Additional updates continued to improve the meta-validation query accuracy, which reached 0.7848 ± 0.0205 after five steps, 0.7925 ± 0.0196 after seven steps, and 0.7976 ± 0.0177 after ten steps. Thus, increasing the adaptation depth from two to ten steps improved the query accuracy by 0.0469. However, the marginal gain gradually diminished, with an improvement of only 0.0051 from seven to ten steps. Meanwhile, the support–query accuracy gap increased from 0.0529 after two steps to 0.0862 after five steps and 0.1121 after ten steps. These results indicate that the original two-step configuration provided effective but incomplete task adaptation. Further updates continued to improve query performance but increasingly favored support-set fitting and produced progressively smaller generalization gains.

The gradient-alignment results provided no indication that systematic inner-loop gradient conflict was a major factor contributing to the lower performance of MAML. The mean cosine similarities between the support and meta-validation query gradients were 0.9411 for the complete task model and 0.9012 for the spectral backbone, and no negative gradient alignment was observed across the evaluated episodes. These positive similarities indicate that the update directions estimated from the support samples were generally aligned with the directions that would reduce the meta-validation query loss. Thus, systematic support–query gradient conflict was not evident under the evaluated setting, although other limitations associated with optimization-based adaptation cannot be excluded.

The task-diversity analysis showed that the episodic sampling process provided adequate class-combination coverage. All 165 possible three-way class combinations were covered under each of the five random number generator seeds, producing a coverage rate of 1.0000. The normalized combination entropy was approximately 0.9998, which was close to its maximum value of 1 and indicated a nearly uniform sampling distribution. Therefore, the comparatively lower performance of MAML was unlikely to result primarily from restricted or highly imbalanced sampling of the possible class combinations.

Overall, MAML exhibited stable but relatively gradual adaptation on the soybean spectral data. Increasing the adaptation depth improved meta-validation query accuracy, but the gains gradually diminished and were accompanied by an expanding support–query gap. Together with the gradient-alignment and task-diversity results, this pattern suggests that the lower performance of MAML was not primarily attributable to systematic support–query gradient conflict or inadequate episodic diversity. Because MAML remained below the metric-based methods reported in Table 2, the results further suggest that prototype aggregation and learned relation measurement may provide a more suitable inductive bias than gradient-based task adaptation for distinguishing the subtle and highly correlated spectral distributions examined in this study.

### 3.5. Effectiveness of the Proposed RDSF Module Under the Known-Class Protocol

Under the known-class evaluation protocol, the effectiveness of the proposed RDSF module was evaluated from two complementary perspectives. First, RDSF was embedded into RelationNet, ProtoNet, and MAML to assess its effects on metric-based and optimization-based meta-learning frameworks under the 3-way K-shot 15-query settings. Second, RelationNet and RDSF–RelationNet were compared under different N-way 10-shot 15-query settings to examine whether the effect of RDSF was retained as the number of classes per episode increased.

As shown in Table 2, the RDSF-enhanced models generally achieved better test performance than their corresponding baseline models under most 3-way few-shot settings. For RelationNet, RDSF–RelationNet achieved test accuracies of 0.9072 ± 0.0079, 0.9184 ± 0.0060, and 0.9202 ± 0.0076 under the 5-shot, 10-shot, and 15-shot settings, respectively. Compared with the original RelationNet, the corresponding test accuracies increased from 0.9014 to 0.9072 in the 5-shot setting, from 0.8898 to 0.9184 in the 10-shot setting, and from 0.9145 to 0.9202 in the 15-shot setting. Thus, RDSF consistently increased the mean test accuracy of RelationNet across the three support-set sizes, although the magnitude of improvement varied among the settings.

For ProtoNet, the improvement brought by RDSF was also evident. Under the 3-way 5-shot setting, RDSF-ProtoNet achieved a test accuracy of 0.9044 ± 0.0051, compared with 0.8498 ± 0.0131 for the original ProtoNet. Under the 10-shot and 15-shot settings, RDSF-ProtoNet increased the test accuracy from 0.8862 to 0.9079 and from 0.8965 to 0.9067, respectively. Moreover, the standard deviation of the test accuracy decreased after introducing RDSF in all three shot settings, indicating lower variation in performance across the five dataset partitions. The largest improvement for ProtoNet occurred in the 5-shot setting, suggesting that the additional derivative-guided representation was particularly beneficial when the support set was smallest.

For MAML, the effect of RDSF was less consistent than that observed for RelationNet and ProtoNet. Under the 3-way 5-shot setting, RDSF-MAML obtained a test accuracy of 0.7314 ± 0.0200, which was slightly lower than the 0.7352 ± 0.0160 obtained by the original MAML. However, under the 10-shot and 15-shot settings, RDSF-MAML increased the test accuracy from 0.7421 to 0.7603 and from 0.7260 to 0.7474, respectively. Therefore, the contribution of RDSF to MAML depended on the support-set size and was less consistent than its contribution to the two metric-based frameworks. This framework-dependent behavior is consistent with the adaptation characteristics examined in Section 3.4.

Among all models in Table 2, RDSF–RelationNet achieved the highest test accuracy under all three known-class 3-way K-shot settings. This consistent ranking suggests that the spectral representation produced by RDSF was particularly compatible with the learned relation metric. However, these overall comparisons do not isolate the contributions of the individual spectral branches or the fusion architecture; these factors are examined through the controlled ablation and architecture comparisons in Section 3.7.

Table 4 further compares RelationNet and RDSF–RelationNet under different N-way 10-shot 15-query settings. As the number of classes increased, each episode involved more inter-class comparisons, increasing the complexity of the classification task. RelationNet achieved test accuracies of 0.8898 ± 0.0201, 0.8996 ± 0.0031, and 0.8595 ± 0.0047 under the 3-way, 5-way, and 11-way settings, respectively, whereas RDSF–RelationNet achieved corresponding accuracies of 0.9184 ± 0.0060, 0.9081 ± 0.0074, and 0.8640 ± 0.0143. Compared with RelationNet, RDSF–RelationNet increased the mean test accuracy by 0.0286, 0.0085, and 0.0045 under the three settings, respectively. The improvement was most evident in the 3-way setting and remained observable in the more complex 5-way and 11-way tasks. In particular, RDSF–RelationNet retained a higher test accuracy in the 11-way setting, in which all 11 soybean varieties were included in each episode. These results show that RDSF remained beneficial across different known-class task difficulties.

Overall, the known-class results in Table 2 and Table 4 show that RDSF consistently increased the mean test accuracy of RelationNet and ProtoNet across all evaluated support-set sizes and increased that of RelationNet across all evaluated N-way settings. In contrast, its effect on MAML varied with the support-set size. These findings support RDSF as an effective spectral representation module for metric-based few-shot classification of predefined soybean varieties, while also showing that its contribution depends partly on the underlying meta-learning framework and task configuration. Generalization to varieties excluded from meta-training is evaluated separately under the strict class-disjoint protocol in Section 3.6.

### 3.6. Class-Disjoint Unseen-Class Evaluation

To complement the original known-class evaluation, an additional strict class-disjoint experiment was conducted to assess generalization to previously unseen soybean varieties, following the protocol described in Section 2.4.2. Table 5 presents the resulting meta-test performance under the 3-way 5-shot, 10-shot, and 15-shot settings. Because the meta-test varieties were completely excluded from meta-training and model selection, these results assess the classification of held-out varieties rather than episodic resampling within a shared label space.

RelationNet achieved meta-test accuracies of 0.8891 ± 0.0135, 0.8953 ± 0.0214, and 0.9019 ± 0.0172 under the 5-shot, 10-shot, and 15-shot settings, respectively. After introducing RDSF, the corresponding accuracies increased to 0.8999 ± 0.0228, 0.8980 ± 0.0185, and 0.9125 ± 0.0300, representing absolute improvements of 0.0108, 0.0027, and 0.0106. Thus, RDSF increased the mean meta-test accuracy of RelationNet across all three support-set sizes, although the magnitude of improvement varied among the settings.

ProtoNet achieved meta-test accuracies of 0.8848 ± 0.0253, 0.9218 ± 0.0147, and 0.9120 ± 0.0192 under the three shot settings, respectively. RDSF-ProtoNet increased these values to 0.9094 ± 0.0080, 0.9224 ± 0.0174, and 0.9199 ± 0.0183, corresponding to absolute improvements of 0.0246, 0.0006, and 0.0079. The largest improvement was observed in the 5-shot setting, where the standard deviation also decreased from 0.0253 to 0.0080. Although the improvements were smaller in the 10-shot and 15-shot settings, RDSF-ProtoNet retained a higher mean meta-test accuracy than ProtoNet in both cases.

For MAML, the effect of RDSF was less consistent. MAML achieved meta-test accuracies of 0.7902 ± 0.0121, 0.8017 ± 0.0112, and 0.8040 ± 0.0102 under the 5-shot, 10-shot, and 15-shot settings, respectively, whereas RDSF-MAML obtained corresponding accuracies of 0.7913 ± 0.0113, 0.7996 ± 0.0093, and 0.8035 ± 0.0095. RDSF therefore produced a marginal improvement of 0.0011 in the 5-shot setting but numerical decreases of 0.0021 and 0.0005 in the 10-shot and 15-shot settings, respectively. These small and inconsistent differences indicate that the improvements observed for RDSF-MAML under some known-class settings were not retained consistently for the held-out varieties. This result further supports the framework-dependent behavior identified in the known-class evaluation and the MAML diagnostic analysis in Section 3.4.

Overall, under the evaluated strict class-disjoint protocol, RDSF consistently increased the meta-test accuracy of both metric-based frameworks across all three support-set sizes. The most pronounced improvement was obtained by RDSF-ProtoNet in the 5-shot setting. In contrast, RDSF did not consistently improve MAML, indicating that its contribution depended partly on the underlying meta-learning mechanism. Together with the known-class results in Section 3.5, these findings show that RDSF provided consistent benefits for ProtoNet and RelationNet under both predefined-class and held-out-class conditions, while its effect on gradient-based adaptation was more dependent on the evaluation setting.

### 3.7. Ablation and Architecture Comparison of the RDSF Module

To further investigate the contribution of different spectral components in the proposed RDSF module, an ablation experiment was conducted under the representative known-class 3-way 10-shot 15-query RelationNet setting. The ablation results are shown in Table 6. In addition to the original RelationNet, four RDSF-based variants were compared: RDSF-Raw, RDSF-Raw+D1, RDSF-Raw+D2, and RDSF-Raw+D1+D2. The RDSF-Raw variant used only the raw spectral branch within the RDSF-style backbone, whereas the original RelationNet used the original SpectralCNN embedding network. Therefore, RelationNet served as the baseline framework, while RDSF-Raw served as an architecture-controlled variant for distinguishing the effect of the RDSF-style backbone from the additional contributions of the derivative branches.

Compared with the original RelationNet, RDSF-Raw increased the test accuracy from 0.8898 ± 0.0201 to 0.8981 ± 0.0106. This comparison shows that replacing the original SpectralCNN backbone with the RDSF-style raw branch produced an absolute improvement of 0.0083 even without derivative inputs. However, because the two models used different embedding architectures, this result alone does not establish the contribution of derivative information. The derivative branches were therefore evaluated by comparing RDSF-Raw with the remaining RDSF variants.

After introducing the first-order derivative branch, RDSF-Raw+D1 achieved a test accuracy of 0.9084 ± 0.0121, representing improvements of 0.0186 over RelationNet and 0.0103 over RDSF-Raw. Because D1 emphasizes local slope variations along the wavelength dimension, this result supports its role as a complementary representation to the raw spectrum. RDSF-Raw+D2 achieved a slightly higher test accuracy of 0.9107 ± 0.0066, corresponding to improvements of 0.0209 over RelationNet and 0.0126 over RDSF-Raw. This result similarly supports a complementary contribution from the curvature-related variations represented by D2. The lower test-accuracy standard deviation of RDSF-Raw+D2 also indicates lower variation across the five dataset partitions than that observed for RelationNet, RDSF-Raw, and RDSF-Raw+D1.

Among all ablation variants, the complete RDSF module, namely RDSF-Raw+D1+D2, achieved the highest validation and test accuracies, reaching 0.9416 ± 0.0049 and 0.9184 ± 0.0060, respectively. Compared with RelationNet, the complete module increased the test accuracy by 0.0286. It also outperformed RDSF-Raw, RDSF-Raw+D1, and RDSF-Raw+D2 by 0.0203, 0.0100, and 0.0077, respectively. The additional improvement obtained when D1 and D2 were used together supports the complementary contributions of the raw, first-order derivative, and second-order derivative representations within the residual fusion module.

**Figure 4:**
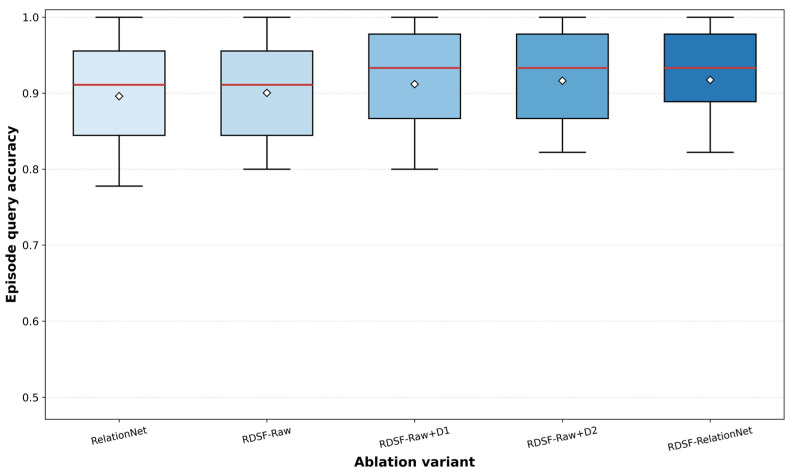
Episode-level query-accuracy distributions of RelationNet and the RDSF ablation variants under the known-class 3-way 10-shot 15-query setting. The boxes represent the interquartile ranges, the red lines indicate the medians, the white diamonds indicate the means, and the whiskers indicate the 10th and 90th percentiles.

To determine whether the improvements in mean accuracy were consistently observed across different few-shot tasks, episode-level query-accuracy variability was evaluated using 1000 fixed meta-test episodes per dataset partition. As shown in Figure 4, RDSF–RelationNet achieved a higher median episode accuracy than RelationNet (0.9333 versus 0.9111). Its task-wise standard deviation and variance decreased from 0.0823 and 0.0068 to 0.0701 and 0.0049, respectively, while its 10th-percentile accuracy increased from 0.7778 to 0.8178. The improvement in the lower percentile indicates that RDSF–RelationNet also performed better on relatively difficult episodes rather than obtaining its overall advantage from only a small number of favorable tasks. RDSF-Raw+D2 exhibited closely comparable episode-level variability, indicating that the additional reduction in dispersion obtained by combining both derivative branches was modest. Overall, the higher median, higher 10th-percentile accuracy, and reduced dispersion show that the improvement of the complete RDSF module was broadly distributed across the evaluated episodes.

To determine whether residual fusion provided advantages over simpler methods for integrating Raw, D1, and D2, two additional architecture variants were evaluated. In ThreeChannel-RelationNet, the three spectral representations were stacked as three input channels and processed by a single shared convolutional backbone. In Concat-RelationNet, Raw, D1, and D2 were processed by the same independent branches used in RDSF, after which the three 64-dimensional embeddings were directly concatenated into a 192-dimensional feature and projected back to 64 dimensions. RDSF–RelationNet retained the independent branches but integrated their embeddings using the proposed bounded residual formulation. All three variants were trained and evaluated using the same five dataset partitions, known-class 3-way 10-shot 15-query configuration, training settings, and validation-based model-selection procedure. The results are presented in Table 7.

As shown in Table 7, ThreeChannel-RelationNet achieved validation and test accuracies of 0.9105 ± 0.0092 and 0.9092 ± 0.0053, respectively. Although this model contained the fewest trainable parameters, processing Raw, D1, and D2 as equivalent channels through a shared backbone produced lower validation and test accuracies than RDSF–RelationNet. Concat-RelationNet achieved validation and test accuracies of 0.9082 ± 0.0050 and 0.9054 ± 0.0043, respectively. Despite containing the largest number of trainable parameters, direct feature concatenation did not outperform either ThreeChannel-RelationNet or RDSF–RelationNet. Thus, increasing the feature dimension and parameter count through concatenation was not sufficient to improve performance under the evaluated few-shot setting.

RDSF–RelationNet achieved the highest validation accuracy of 0.9416 ± 0.0049 and the highest test accuracy of 0.9184 ± 0.0060. Its test accuracy was 0.0092 higher than that of ThreeChannel-RelationNet and 0.0130 higher than that of Concat-RelationNet. RDSF–RelationNet contained more parameters than the three-channel model but 16,510 fewer parameters than the concatenation model. Therefore, RDSF is not claimed to be the least parameterized architecture. Instead, the controlled comparison shows that its performance cannot be attributed solely to the use of multiple spectral representations, an expanded intermediate feature dimension, or a larger parameter count. Among the evaluated variants, processing the three representations through independent branches and integrating them through bounded residual fusion provided the highest classification accuracy.

Overall, the ablation results support the complementary contributions of the raw, D1, and D2 branches, while the episode-level analysis shows that the improvement of RDSF–RelationNet extended across a broad distribution of few-shot tasks. The architecture comparison further shows that RDSF outperformed both the three-channel shared-backbone architecture and direct feature concatenation. Together, these results support the complete RDSF design as an effective integration strategy for the evaluated known-class few-shot classification setting.

### 3.8. Stability and Effective Contribution of the Learned Fusion Coefficients

To quantitatively examine the behavior of the residual fusion mechanism, the learned derivative coefficients were analyzed under the representative known-class 3-way 10-shot 15-query setting. The coefficient of the Raw branch was fixed at 1.0, whereas the D1 and D2 coefficients, denoted as $\alpha_{1}$ and $\alpha_{2}$, respectively, were learnable. Because the Raw coefficient was fixed and identical across all models, it is not displayed in Table 8. The derivative coefficients were initialized to 0.10 and 0.05 and constrained to the range of 0–0.30. Table 8 reports their values across five independent runs and their variation across individual episodic tasks.

The learned coefficients exhibited framework-dependent behavior. For RDSF–RelationNet, both derivative coefficients reached the predefined upper bound of 0.30 in all five runs. This result indicates that RelationNet consistently assigned the largest derivative weights permitted by the residual constraint. However, the zero across-run standard deviations resulted from boundary saturation and should not be interpreted as evidence that 0.30 represents an unconstrained optimum. Moreover, the equal coefficients do not necessarily imply equal effective contributions from D1 and D2 because their branch-output magnitudes may differ.

For RDSF-ProtoNet, $\alpha_{1}$ and $\alpha_{2}$ were 0.1607 ± 0.0037 and 0.0908 ± 0.0011, respectively. Their relatively small across-run standard deviations indicate that similar coefficient values were obtained across the five independent runs. The larger value of $\alpha_{1}$ shows that ProtoNet assigned a greater residual weight to D1 than to D2, although the coefficient magnitudes alone should not be interpreted as direct measures of physical or chemical importance.

For RDSF-MAML, the coefficients after the original two inner-loop updates were 0.1277 ± 0.0030 for D1 and 0.0612 ± 0.0022 for D2. Unlike RelationNet and ProtoNet, MAML updated these coefficients during task-specific inner-loop adaptation. Nevertheless, their mean task-wise standard deviations were only 0.0018 and 0.0004, respectively, indicating limited episode-to-episode variation under the evaluated setting. As in ProtoNet, MAML assigned a larger residual weight to D1 than to D2.

Because MAML was the only evaluated framework in which the fusion coefficients underwent task-specific inner-loop updates, its effective branch contributions after adaptation were additionally quantified. These contributions were calculated from the relative $L_{2}$ -norms of the Raw, weighted D1, and weighted D2 branch outputs—$F_{Raw}$, $\alpha_{1}F_{D1}$, and $\alpha_{2}F_{D2}$, respectively—before final embedding normalization. The Raw, D1, and D2 branches accounted for 94.80 ± 0.38%, 4.45 ± 0.34%, and 0.75 ± 0.04% of the effective feature magnitude, respectively. Thus, the Raw branch remained the dominant representation after task-specific adaptation, while D1 and D2 acted as smaller residual corrections. Moreover, a small residual contribution may still influence the resulting embedding and classification decision.

Overall, the learned coefficients showed distinct but reproducible framework-dependent patterns: ProtoNet and MAML assigned larger residual weights to D1 than to D2, whereas RelationNet drove both coefficients to the permitted upper bound. The effective contribution analysis further showed that the Raw representation remained dominant after MAML adaptation, with the derivative branches providing limited but stable residual corrections under the evaluated setting.

### 3.9. Visualization and Class-Wise Classification Analysis

**Figure 5:**
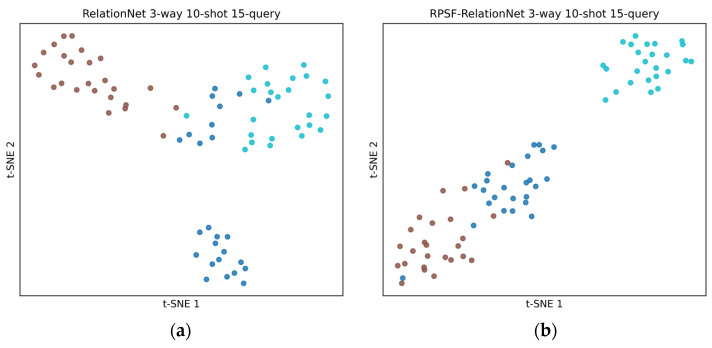

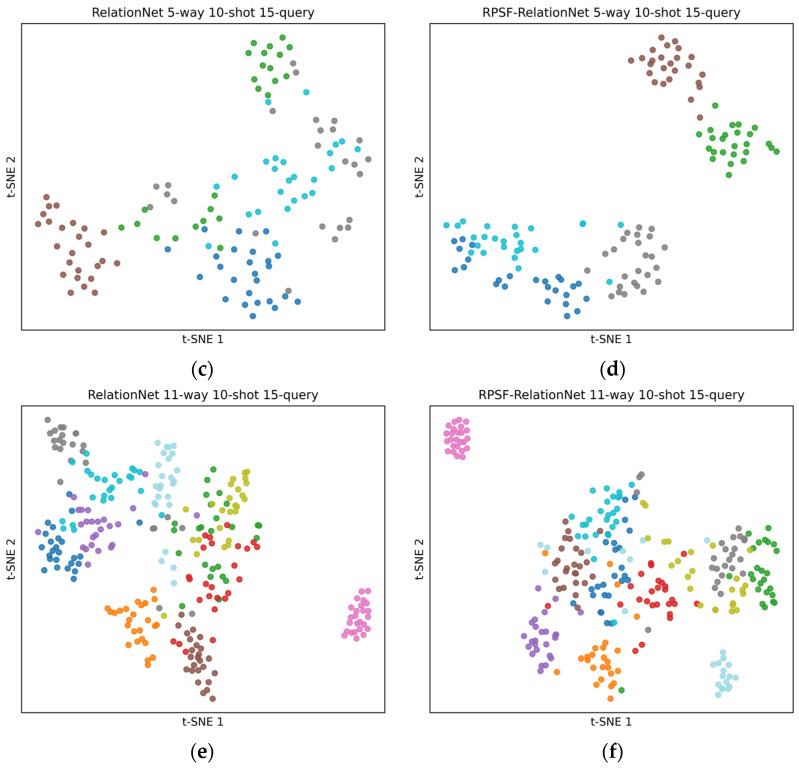
t-SNE visualization of spectral embeddings generated by RelationNet and RDSF–RelationNet under different known-class N-way 10-shot 15-query settings: (**a**,**c**,**e**) RelationNet under the 3-way, 5-way, and 11-way settings, respectively; (**b**,**d**,**f**) RDSF–RelationNet under the corresponding 3-way, 5-way, and 11-way settings, respectively. Different colors represent different seed varieties.

To qualitatively examine the organization of the learned spectral embeddings, t-distributed stochastic neighbor embedding (t-SNE) was applied to the features generated by RelationNet and RDSF–RelationNet under the known-class 3-way, 5-way, and 11-way 10-shot 15-query settings. The resulting two-dimensional projections are shown in Figure 5. Figure 5a,c,e presents the embeddings generated by RelationNet under the three respective N-way settings, whereas Figure 5b,d,f presents the corresponding embeddings generated by RDSF–RelationNet. Because t-SNE is a nonlinear dimensionality-reduction method that may alter distances in the original embedding space, these visualizations were used only for qualitative comparison of local clustering and class overlap and were not treated as direct quantitative evidence of classification performance.

Under the 3-way setting, both RelationNet and RDSF–RelationNet formed distinguishable class distributions. In Figure 5a, the embeddings generated by RelationNet showed separation among the three selected soybean varieties, although the distributions of some samples were relatively scattered. In comparison, Figure 5b shows that RDSF–RelationNet produced more compact local distributions and clearer separation among several projected class groups. This visual pattern suggests that the embeddings generated by RDSF–RelationNet were more consistently organized for the selected 3-way tasks.

When the task difficulty increased to the 5-way setting, greater overlap among the projected class distributions was observed. As shown in Figure 5c, the embeddings generated by RelationNet were relatively dispersed, with partial overlap among several classes. By contrast, Figure 5d shows that RDSF–RelationNet generated more compact and better-separated projected clusters for most of the displayed classes. The difference between the two visualizations was particularly apparent under this setting and was consistent with the quantitative results in Table 4, where RDSF–RelationNet achieved a higher test accuracy than RelationNet (0.9081 versus 0.8996).

Under the 11-way setting, all soybean varieties were included in each episode, making the classification task more complex. As shown in Figure 5e, the RelationNet embeddings exhibited substantial class mixing, particularly in the central region of the projection. Although several varieties formed relatively independent clusters, the overall class boundaries were less distinct than those observed under the lower-way settings. Figure 5f shows that partial overlap also remained in the RDSF–RelationNet embeddings, reflecting the difficulty of simultaneously distinguishing all 11 soybean varieties using limited support samples. Nevertheless, several projected class groups appeared more locally compact after introducing RDSF. This observation is consistent with the test results in Table 4, where RDSF–RelationNet retained a slightly higher accuracy than RelationNet under the 11-way setting (0.8640 versus 0.8595).

Overall, the t-SNE projections provide a qualitative indication that RDSF–RelationNet generally produced more compact local class structures than RelationNet, particularly under the 5-way setting. As the number of classes increased to 11, considerable overlap remained for both models, indicating that RDSF did not eliminate the difficulty arising from highly similar spectral distributions. These visualization patterns are consistent with the quantitative performance improvements reported in Table 4.

**Figure 6:**
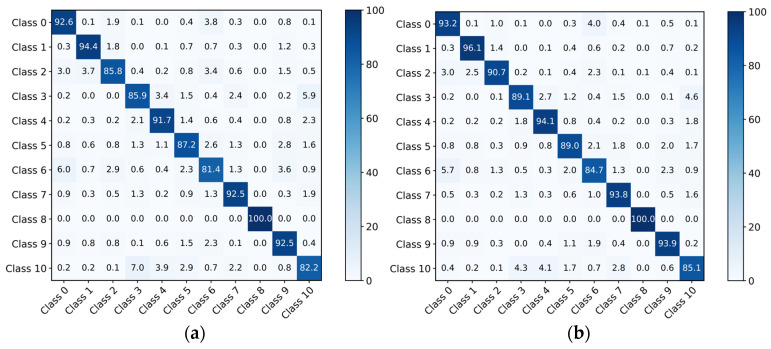
Row-normalized confusion matrices of (**a**) RelationNet and (**b**) RDSF–RelationNet under the known-class 3-way 10-shot 15-query setting. Predictions from 1000 fixed meta-test episodes in each of the five independent dataset partitions were mapped to the original 11 soybean variety labels, row-normalized, and averaged across partitions. Values are expressed as percentages, and each diagonal value represents the recall of the corresponding class.

To complement the qualitative t-SNE analysis with class-wise classification results, Figure 6 presents the row-normalized confusion matrices of RelationNet and RDSF–RelationNet under the representative known-class 3-way 10-shot 15-query setting. Predictions were collected from 1000 fixed meta-test episodes in each of the five independent dataset partitions. Because each episode contained only three of the 11 soybean varieties, the episodic predictions were mapped back to their original variety labels before aggregation. The confusion matrices were then row-normalized and averaged across the five partitions. Consequently, each row represents the prediction distribution for a true class, and each diagonal value represents the recall of the corresponding soybean variety.

Both models exhibited clear diagonal dominance, indicating that most samples were assigned to their correct varieties across the aggregated episodes. Class 8 achieved a recall of 100.0% for both models, suggesting that this variety was consistently distinguished from the other varieties included in the same episodes. Compared with RelationNet, RDSF–RelationNet increased the recall of most classes, with particularly clear improvements for Classes 2, 6, and 10. These class-wise changes are consistent with the higher overall test accuracy achieved by RDSF–RelationNet under the same experimental setting.

Despite these improvements, residual inter-class confusion remained. For RDSF–RelationNet, the principal errors involved Class 6 being predicted as Class 0 and Class 10 being predicted as Classes 3 and 4. These patterns indicate that some soybean varieties retained similar spectral representations even after derivative-guided residual fusion. The confusion matrices therefore provide class-specific information that is not directly available from the overall accuracy or the two-dimensional t-SNE projections.

Taken together, the t-SNE projections and confusion matrices provide complementary views of model performance. The t-SNE results qualitatively illustrate the organization of the learned embeddings under different task difficulties, whereas the confusion matrices quantitatively identify class-wise recall and the principal remaining misclassification patterns. Both analyses are consistent with the higher test accuracy of RDSF–RelationNet reported in Table 4, while neither analysis alone establishes a specific physical or chemical mechanism underlying the improvement.

## 4. Discussion

Accurate identification of soybean seed varieties is important for seed purity testing, variety authentication, germplasm management, and quality control. Because different varieties may have similar external morphology, manual inspection can be subjective and unsuitable for rapid screening. Hyperspectral imaging provides a non-destructive and information-rich alternative by capturing continuous spectral responses associated with seed composition, moisture, surface structure, and light-scattering properties. Previous studies have demonstrated the potential of combining hyperspectral imaging with machine learning or deep learning for seed analysis. DAFFnet used dual-attention feature fusion for soybean variety classification [32], while SUnSeT applied spectral unmixing to soybean seed phenotyping [33]. Recent maize studies have also used multimodal fusion and residual one-dimensional CNNs for seed variety identification [34,35], highlighting the importance of spectral representation learning. However, most existing studies were conducted under conventional supervised-learning conditions with comparatively abundant labeled samples. In contrast, the present study investigated an 11-class soybean variety classification task under few-shot conditions. The test accuracies of SVC and CNN, reaching 0.8890 ± 0.0124 and 0.8857 ± 0.0091, respectively, established the baseline separability of the extracted one-dimensional spectra. RDSF–RelationNet achieved a test accuracy of 0.9184 ± 0.0060 under the known-class 3-way 10-shot 15-query setting and remained competitive under more complex known-class N-way tasks. The strict class-disjoint evaluation further provided evidence that the evaluated meta-learning models could classify varieties excluded from meta-training under the predefined class partition. Collectively, these results extend HSI-based seed variety classification to limited-sample conditions and establish the context for evaluating derivative-guided spectral representation.

Few-shot learning is particularly relevant to hyperspectral seed classification because image acquisition, variety-label confirmation, and balanced dataset construction require considerable time and labor. However, few studies have specifically investigated few-shot hyperspectral seed classification, and most existing methods have focused on spectral–spatial information from remote-sensing images. These methods commonly use spectral–spatial patches to exploit spectral signatures together with spatial neighborhoods, textures, and local structures. Recent approaches have introduced spectral–spatial graph convolution, domain attention, and lightweight dual-branch meta-learning for small-sample HSI classification [36,37,38], while frequency-aware augmentation, graph wavelet convolution, and cross-domain adaptation have been used to improve few-shot or cross-domain generalization [39,40]. Unlike these studies, the present work used the one-dimensional mean reflectance spectrum extracted from each soybean seed rather than the complete spectral–spatial image cube. This representation provides a compact input for seed-level screening and reduces the computational complexity of full-cube modeling. However, without spatial texture and morphological cues, classification depends primarily on subtle differences among highly similar spectral curves, placing greater demands on spectral representation learning. RDSF was therefore developed to integrate the raw spectrum and its first- and second-order derivatives through residual fusion. The results showed that these derivative-guided representations provided complementary information for few-shot soybean variety classification when spatial image features were not directly used.

The performance of RDSF depended on both the evaluation protocol and the underlying meta-learning framework. Under the known-class protocol, RDSF consistently increased the mean test accuracies of ProtoNet and RelationNet across all evaluated shot settings, indicating that derivative-guided spectral fusion was useful when the candidate varieties were predefined but only a small labeled support set was available. These improvements were retained for both metric-based frameworks under the strict class-disjoint protocol, suggesting that the benefit of RDSF was not restricted to episodic classification within a shared label space. The class-disjoint gains were generally moderate, which may reflect the fact that D1 and D2 are deterministic transformations of the raw spectrum rather than independent information sources, while convolutional encoders may already capture part of the corresponding local variation. The relatively high baseline accuracies also left limited room for absolute improvement. The different behaviors of the three frameworks further indicate that the contribution of RDSF was influenced by the task-classification mechanism. RelationNet provided the most consistently competitive baseline performance, and RDSF–RelationNet achieved the highest test accuracy under all three known-class 3-way K-shot settings. Its learned nonlinear relation function may be advantageous for modeling the subtle similarities between class and query representations. ProtoNet also benefited consistently from RDSF, possibly because prototype aggregation reduces the influence of individual support-sample variability and provides stable class-level representations, consistent with the use of prototype learning in recent few-shot HSI studies [41,42]. In contrast, RDSF did not consistently improve MAML under either protocol. The diagnostic analysis provided no indication that systematic support–query gradient conflict or inadequate class-combination diversity was a major factor in this behavior: the support and meta-validation query gradients showed strong positive alignment, and all possible three-way class combinations were covered with an approximately uniform sampling distribution. Increasing the inner-loop adaptation depth improved query accuracy, but the gains progressively diminished while the support–query gap expanded. These observations suggest that prototype aggregation and learned relation measurement may provide a more suitable inductive bias than the evaluated gradient-based adaptation procedure for the highly similar one-dimensional soybean spectra. However, they do not identify a single definitive cause of the lower MAML performance or exclude the possibility that alternative adaptation configurations could improve its behavior.

Beyond the evaluation protocol and meta-learning framework, the effect of RDSF was also influenced by task difficulty. As the number of classes increased, each episode involved more inter-class comparisons and more complex decision boundaries. RDSF–RelationNet achieved higher mean test accuracy than RelationNet under the known-class 3-way, 5-way, and 11-way settings, showing that the benefit of derivative-guided fusion was retained across different task complexities. However, the absolute improvements decreased from 0.0286 in the 3-way setting to 0.0085 and 0.0045 in the 5-way and 11-way settings, respectively. The smaller gain in the 11-way task indicates that derivative-based enhancement could not eliminate the intrinsic overlap among all 11 varieties when they were classified simultaneously. The t-SNE projections were consistent with this interpretation. RDSF–RelationNet generally produced more compact local class structures than RelationNet, particularly in the 5-way setting, whereas considerable overlap remained for both models in the 11-way projection. Because t-SNE is a nonlinear two-dimensional projection, these patterns provide only qualitative evidence and should not be interpreted as direct measurements of separability in the original embedding space. The row-normalized confusion matrices provided complementary class-wise evidence: RDSF–RelationNet improved the recall of most classes, particularly Classes 2, 6, and 10, while Class 8 was consistently classified correctly by both models. The remaining errors were concentrated mainly between Class 6 and Class 0 and between Class 10 and Classes 3–4, indicating that some varieties retained highly similar spectral representations after fusion. Together, the N-way results, t-SNE projections, and confusion matrices show that RDSF improved spectral discrimination under different task difficulties but did not fully resolve the challenge of separating all spectrally similar varieties.

The observed improvements can be interpreted from both spectral and architectural perspectives. The raw spectrum retains the complete reflectance profile, whereas D1 and D2 emphasize local slope and curvature variations, respectively. Several wavelength regions observed in the spectral profiles have previously been associated with O–H-, C–H-, and N–H-related absorption in water, lipids, carbohydrates, and proteins [28,29,30,31]. However, D1 and D2 remain deterministic mathematical transformations of the raw spectrum, and the present study did not directly measure these chemical constituents or determine which wavelength regions influenced the model predictions. The established spectroscopic assignments therefore provide context for the measured spectra but do not prove that RDSF relied on specific biochemical absorption features. The ablation analysis nevertheless provided direct evidence that the derivative representations contributed to classification performance. RDSF-Raw outperformed the original RelationNet, showing that part of the improvement was associated with the RDSF-style raw branch. Because these two variants used different embedding backbones, this comparison alone cannot establish the contribution of derivative information. However, adding either D1 or D2 to the RDSF-Raw variant produced further improvements, and the complete Raw+D1+D2 configuration achieved the highest validation and test accuracies among the ablation variants. These results support complementary contributions from the derivative branches beyond the effect of backbone replacement. The controlled architecture comparison further showed that RDSF–RelationNet outperformed both the three-channel shared-backbone architecture and the multi-branch direct-concatenation architecture. Concat-RelationNet contained more trainable parameters than RDSF–RelationNet but achieved lower validation and test accuracies, indicating that increasing feature dimensionality and parameter count was not sufficient to explain the improvement. Conversely, ThreeChannel-RelationNet contained substantially fewer parameters, and RDSF should therefore not be regarded as the least parameterized alternative. Taken together, the ablation and architecture comparisons support independent branch processing followed by bounded residual fusion as the most effective integration strategy among the evaluated variants, although they do not establish it as theoretically optimal or superior to all possible fusion architectures.

The episode-level and coefficient analyses further clarified the reliability and internal behavior of the residual fusion mechanism. Compared with RelationNet, RDSF–RelationNet increased the median episode accuracy from 0.9111 to 0.9333 and the 10th-percentile accuracy from 0.7778 to 0.8178, while reducing the task-wise standard deviation from 0.0823 to 0.0701. The improvement in the lower percentile indicates that the overall gain was not driven solely by a small number of relatively easy episodes. However, RDSF-Raw+D2 showed closely comparable episode-level variability, indicating that the additional stability improvement obtained by combining both derivative branches was modest, even though the complete module achieved the highest mean test accuracy. The learned coefficients showed additional framework-dependent behavior. ProtoNet and MAML assigned larger residual weights to D1 than to D2, whereas RelationNet drove both derivative coefficients to the predefined upper bound of 0.30. This boundary saturation indicates that RelationNet preferred the strongest derivative corrections permitted by the implemented constraint but does not establish 0.30 as an unconstrained optimum. For RelationNet and ProtoNet, the coefficients were globally shared and were not updated during individual meta-test episodes; their zero task-wise variation was therefore structural rather than empirical evidence of exceptional stability. In MAML, the coefficients underwent task-specific adaptation but exhibited only small task-wise standard deviations. The corresponding norm-based analysis attributed 94.80%, 4.45%, and 0.75% of the effective feature magnitude to Raw, D1, and D2, respectively, showing that the Raw branch remained dominant after MAML adaptation while the derivative branches acted as smaller residual corrections.

From an application perspective, the proposed RDSF module provides a practical spectral representation strategy for soybean seed variety classification under limited labeled samples. The known-class protocol represents scenarios in which the candidate varieties are predefined but only a small number of labeled reference seeds are available, whereas the class-disjoint protocol approximates adaptation to a held-out variety after a limited support set has been collected. These scenarios are relevant to seed testing, variety authentication, and germplasm management, where constructing large balanced datasets for every target variety may be impractical. RDSF can be integrated into different episodic frameworks without changing their fundamental training and inference procedures, although its performance benefits were most consistent for ProtoNet and RelationNet. In addition, representing each seed by its one-dimensional mean spectrum reduces input and computational complexity relative to full spectral–spatial cube modeling and may facilitate the future development of rapid seed-level screening systems.

Nevertheless, this study has several limitations. First, the dataset contained only 11 soybean varieties, and the strict class-disjoint evaluation used five varieties for meta-training, three for meta-validation, and three for meta-testing under a fixed class partition. The reported results therefore provide evidence of held-out-variety classification under this predefined partition but do not represent all possible combinations of training and unseen varieties. Second, only one-dimensional mean spectra were used, and spatial texture, seed morphology, and within-seed spectral variation from the original hyperspectral images were not directly exploited. Third, all spectra were acquired using a single hyperspectral imaging system under controlled conditions. The study did not evaluate domain shifts associated with different sensors, acquisition periods, production regions, seed batches, moisture conditions, storage histories, or illumination environments, and RDSF should therefore not be assumed to be inherently domain invariant. Future work should therefore expand the dataset to include more varieties and multiple class-level partitions, evaluate cross-batch, cross-region, cross-year, and cross-device generalization, and investigate calibration transfer or domain-adaptive meta-learning. Further studies should also examine the integration of mean spectra with spatial and morphological information, systematically compare the moving-average procedure with Savitzky–Golay smoothing and differentiation under identical experimental settings, and combine wavelength-importance analysis with chemical measurements to improve the spectroscopic interpretability of RDSF.

## 5. Conclusions

In this study, an RDSF module was developed for few-shot classification using one-dimensional hyperspectral spectra from 11,000 individual soybean seeds representing 11 varieties. RDSF processes the raw spectrum and its first- and second-order derivatives through separate branches and combines them through residual fusion, with the derivative representations providing complementary slope- and curvature-related information. As a plug-and-play representation module, RDSF was integrated into ProtoNet, RelationNet, and MAML and evaluated under both known-class and strict class-disjoint unseen-class protocols. Under the representative known-class 3-way 10-shot 15-query setting, RDSF increased the test accuracy of RelationNet from 0.8898 ± 0.0201 to 0.9184 ± 0.0060. Under the class-disjoint protocol, RDSF consistently improved ProtoNet and RelationNet across all evaluated shot settings, with the largest gain observed for the 5-shot ProtoNet, from 0.8848 ± 0.0253 to 0.9094 ± 0.0080. In contrast, RDSF did not consistently improve MAML, indicating that its contribution depended partly on the adaptation mechanism of the underlying framework.

The ablation results supported the complementary contributions of the Raw, D1, and D2 branches, with the complete RDSF configuration achieving the highest validation and test accuracies among the evaluated ablation variants. The controlled architecture comparison further showed that residual fusion outperformed both the three-channel shared-backbone architecture and direct feature concatenation, while the episode-level analysis indicated that the improvement extended across a broad distribution of few-shot tasks. Overall, these findings support RDSF as an effective spectral representation module for metric-based few-shot classification of soybean seed varieties under the evaluated known-class and class-disjoint conditions. Future work should evaluate larger variety pools, multiple class partitions, seed batches, production regions, acquisition years, and imaging devices, while further investigating spectral–spatial integration, alternative derivative preprocessing methods, and wavelength-level spectroscopic interpretability.

## Figures and Tables

**Table 1:** Classification performance of conventional supervised models across five dataset partitions.

Model	Train ACC	Val ACC	Test ACC
SVC	0.9482 ± 0.0034	0.8865 ± 0.0114	**0.8890 ± 0.0124**
**CNN**	**0.9644 ± 0.0078**	**0.8923 ± 0.0054**	0.8857 ± 0.0091
ResNet	0.9026 ± 0.0152	0.8256 ± 0.0095	0.8186 ± 0.0124
Transformer	0.8065 ± 0.0178	0.7327 ± 0.0143	0.7245 ± 0.0169
LSTM	0.6963 ± 0.0367	0.6254 ± 0.0192	0.6258 ± 0.0285
CNN–Transformer	0.8604 ± 0.0225	0.7933 ± 0.0209	0.7900 ± 0.0121
CNN-LSTM	0.7934 ± 0.0282	0.7249 ± 0.0275	0.7224 ± 0.0356

Note: Values are reported as the mean accuracy ± standard deviation across five independent dataset partitions. The highest value in each column is highlighted in bold.

**Table 2:** Few-shot classification performance of baseline and RDSF-enhanced meta-learning models under the known-class 3-way K-shot 15-query settings.

K-Shot	Model	Train ACC	Val ACC	Test ACC
5-shot	RelationNet	0.9511 ± 0.0507	**0.9259 ± 0.0086**	0.9014 ± 0.0070
	RDSF–RelationNet	**0.9867 ± 0.0199**	0.9251 ± 0.0026	**0.9072 ± 0.0079**
	ProtoNet	0.8933 ± 0.0397	0.9178 ± 0.0059	0.8498 ± 0.0131
	RDSF-ProtoNet	0.9467 ± 0.0337	0.9035 ± 0.0075	0.9044 ± 0.0051
	MAML	0.7443 ± 0.0207	0.7479 ± 0.0201	0.7352 ± 0.0160
	RDSF-MAML	0.7171 ± 0.0084	0.7665 ± 0.0127	0.7314 ± 0.0200
10-shot	RelationNet	0.9035 ± 0.0081	0.9246 ± 0.0026	0.8898 ± 0.0201
	RDSF–RelationNet	0.9365 ± 0.0069	**0.9416 ± 0.0049**	**0.9184 ± 0.0060**
	ProtoNet	0.9333 ± 0.0416	0.9378 ± 0.0108	0.8862 ± 0.0094
	RDSF-ProtoNet	**0.9822 ± 0.0186**	0.9215 ± 0.0055	0.9079 ± 0.0053
	MAML	0.7637 ± 0.0171	0.7484 ± 0.0226	0.7421 ± 0.0253
	RDSF-MAML	0.7742 ± 0.0045	0.7757 ± 0.0221	0.7603 ± 0.0155
15-shot	RelationNet	0.9422 ± 0.0579	**0.9376 ± 0.0034**	0.9145 ± 0.0062
	RDSF–RelationNet	**0.9956 ± 0.0099**	0.9365 ± 0.0034	**0.9202 ± 0.0076**
	ProtoNet	0.8845 ± 0.0243	0.9304 ± 0.0025	0.8965 ± 0.0301
	RDSF-ProtoNet	0.9645 ± 0.0122	0.9292 ± 0.0042	0.9067 ± 0.0044
	MAML	0.7629 ± 0.0201	0.7567 ± 0.0189	0.7260 ± 0.0238
	RDSF-MAML	0.7618 ± 0.0295	0.8024 ± 0.0168	0.7474 ± 0.0133

Note: Values are reported as the mean accuracy ± standard deviation across five independent dataset partitions. The highest value for each metric within each K-shot setting is highlighted in bold.

**Table 3:** Inner-loop adaptation trajectory of MAML on fixed meta-validation episodes under the known-class 3-way 10-shot 15-query setting.

Inner-Loop Steps	Support ACC	Query ACC	Support–Query Gap
0	0.3359 ± 0.0196	0.3350 ± 0.0174	0.0009
1	0.7101 ± 0.0202	0.6781 ± 0.0219	0.0320
2	0.8036 ± 0.0162	0.7507 ± 0.0214	0.0529
3	0.8381 ± 0.0172	0.7692 ± 0.0222	0.0689
5	0.8710 ± 0.0160	0.7848 ± 0.0205	0.0862
7	0.8909 ± 0.0138	0.7925 ± 0.0196	0.0984
10	0.9097 ± 0.0130	0.7976 ± 0.0177	0.1121

Note: Values are reported as the mean ± standard deviation across five independent dataset partitions, with 200 fixed meta-validation episodes averaged within each partition. The same support/query samples and task-head initialization were used across all inner-loop steps within each episode. The inner-loop learning rate was fixed at 0.01. The support–query gap was calculated as the difference between the mean support and meta-validation query accuracies. This post hoc diagnostic analysis used MAML backbones obtained under the original two-step training protocol and does not represent final meta-test performance.

**Table 4:** Classification performance of RelationNet and RDSF–RelationNet under different known-class N-way 10-shot 15-query settings.

N-Way	Model	Train ACC	Val ACC	Test ACC
3-way	RelationNet	0.9035 ± 0.0081	0.9246 ± 0.0026	0.8898 ± 0.0201
	**RDSF–RelationNet**	**0.9365 ± 0.0069**	**0.9416 ± 0.0049**	**0.9184 ± 0.0060**
5-way	RelationNet	0.9259 ± 0.0051	0.9148 ± 0.0034	0.8996 ± 0.0031
	**RDSF–RelationNet**	**0.9657 ± 0.0087**	**0.9189 ± 0.0053**	**0.9081 ± 0.0074**
11-way	RelationNet	0.9455 ± 0.0121	**0.8711 ± 0.0094**	0.8595 ± 0.0047
	**RDSF–RelationNet**	**0.9557 ± 0.0186**	0.8696 ± 0.0075	**0.8640 ± 0.0143**

Note: Values are reported as the mean accuracy ± standard deviation across five independent dataset partitions. For each N-way setting, the higher mean accuracy between the two models for each data subset is highlighted in bold.

**Table 5:** Classification performance under the strict class-disjoint unseen-class evaluation protocol.

Method	3-Way 5-Shot	3-Way 10-Shot	3-Way 15-Shot
RelationNet	0.8891 ± 0.0135	0.8953 ± 0.0214	0.9019 ± 0.0172
RDSF–RelationNet	0.8999 ± 0.0228	0.8980 ± 0.0185	0.9125 ± 0.0300
ProtoNet	0.8848 ± 0.0253	0.9218 ± 0.0147	0.9120 ± 0.0192
RDSF-ProtoNet	**0.9094 ± 0.0080**	**0.9224 ± 0.0174**	**0.9199 ± 0.0183**
MAML	0.7902 ± 0.0121	0.8017 ± 0.0112	0.8040 ± 0.0102
RDSF-MAML	0.7913 ± 0.0113	0.7996 ± 0.0093	0.8035 ± 0.0095

Note: Values are reported as the mean meta-test accuracy ± standard deviation over five independent runs. The meta-training, meta-validation, and meta-test classes were strictly disjoint. The highest mean accuracy in each shot setting is highlighted in bold.

**Table 6:** Ablation analysis of the RDSF module under the known-class 3-way 10-shot 15-query RelationNet setting.

Model Variant	Backbone/Input Configuration	Raw	D1	D2	Train ACC	Val ACC	Test ACC	∆Test ACC
RelationNet	Original SpectralCNN backbone	√	×	×	0.9035 ± 0.0081	0.9246 ± 0.0026	0.8898 ± 0.0201	-
RDSF-Raw	RDSF-style raw branch only	√	×	×	0.9154 ± 0.0152	0.9298 ± 0.0020	0.8981 ± 0.0106	+0.0083
RDSF-Raw+D1	Raw branch + first-derivative branch	√	√	×	0.9225 ± 0.0078	0.9391 ± 0.0040	0.9084 ± 0.0121	+0.0186
RDSF-Raw+D2	Raw branch + second-derivative branch	√	×	√	0.9430 ± 0.0081	0.9405 ± 0.0041	0.9107 ± 0.0066	+0.0209
RDSF–RelationNet	Full RDSF module	√	√	√	0.9365 ± 0.0069	**0.9416 ± 0.0049**	**0.9184 ± 0.0060**	**+0.0286**

Note: Values are reported as the mean accuracy ± standard deviation across five independent dataset partitions. ∆Test ACC denotes the absolute change in mean test accuracy relative to the original RelationNet. The highest validation and test accuracies and the largest test-accuracy improvement are highlighted in bold. √ represents usage, × represents removal.

**Table 7:** Controlled comparison of spectral integration architectures under the known-class 3-way 10-shot 15-query RelationNet setting.

Model	Fusion Strategy	Parameters (M)	Val ACC	Test ACC
ThreeChannel-RelationNet	Three-channel shared backbone	0.8631	0.9105 ± 0.0092	0.9092 ± 0.0053
Concat-RelationNet	Multi-branch feature concatenation	1.4752	0.9082 ± 0.0050	0.9054 ± 0.0043
RDSF–RelationNet	Bounded residual fusion	1.4587	**0.9416 ± 0.0049**	**0.9184 ± 0.0060**

Note: Accuracy values are reported as the mean ± standard deviation across five independent dataset partitions. Trainable parameters are reported in millions (M). The highest validation and test accuracies are highlighted in bold.

**Table 8:** Learned fusion coefficients and their task-wise stability under the representative known-class 3-way 10-shot 15-query setting.

Model	$\alpha_{1}$(D1)	$\alpha_{2}$(D2)
RDSF–RelationNet	0.3000 ± 0.0000 (0.0000)	0.3000 ± 0.0000 (0.0000)
RDSF-ProtoNet	0.1607 ± 0.0037 (0.0000)	0.0908 ± 0.0011 (0.0000)
RDSF-MAML	0.1277 ± 0.0030 (0.0018)	0.0612 ± 0.0022 (0.0004)

Note: Values outside parentheses are reported as the mean ± standard deviation across five independent runs, while values in parentheses denote the mean task-wise standard deviation. For RDSF–RelationNet and RDSF-ProtoNet, the coefficients were globally shared and were not updated within individual meta-test episodes; therefore, their task-wise standard deviations were structurally zero.

## Data Availability

The original contributions presented in this study are included in the article. Further inquiries can be directed to the corresponding authors.
